# A Review of Lawsuits Related to Point-of-Care Emergency Ultrasound Applications

**DOI:** 10.5811/westjem.2014.11.23592

**Published:** 2014-12-12

**Authors:** Lori Stolz, Kathleen M. O’Brien, Marc L. Miller, Nicole D. Winters-Brown, Michael Blaivas, Srikar Adhikari

**Affiliations:** * University of Arizona, Department of Emergency Medicine, Tucson, Arizona; † Massachusetts General Hospital, Division of Global Health and Human Rights, Boston, Massachusetts; ‡ University of Arizona, James E. Roger College of Law, Tucson, Arizona; § American University, Washington College of Law, Washington DC; || St. Francis Hospital, Department of Emergency Medicine, Columbus, Georgia

## Abstract

**Introduction:**

New medical technology brings the potential of lawsuits related to the usage of that new technology. In recent years the use of point-of-care (POC) ultrasound has increased rapidly in the emergency department (ED). POC ultrasound creates potential legal risk to an emergency physician (EP) either using or not using this tool. The aim of this study was to quantify and characterize reported decisions in lawsuits related to EPs performing POC ultrasound.

**Methods:**

We conducted a retrospective review of all United States reported state and federal cases in the Westlaw database. We assessed the full text of reported cases between January 2008 and December 2012. EPs with emergency ultrasound fellowship training reviewed the full text of each case. Cases were included if an EP was named, the patient encounter was in the emergency department, the interpretation or failure to perform an ultrasound was a central issue and the application was within the American College of Emergency Physician (ACEP) ultrasound core applications. In order to assess deferred risk, cases that involved ultrasound examinations that could have been performed by an EP but were deferred to radiology were included.

**Results:**

We identified five cases. All reported decisions alleged a failure to perform an ultrasound study or a failure to perform it in a timely manner. All studies were within the scope of emergency medicine and were ACEP emergency ultrasound core applications. A majority of cases (n=4) resulted in a patient death. There were no reported cases of failure to interpret or misdiagnoses.

**Conclusion:**

In a five-year period from January 2008 through December 2012, five malpractice cases involving EPs and ultrasound examinations that are ACEP core emergency ultrasound applications were documented in the Westlaw database. All cases were related to failure to perform an ultrasound study or failure to perform a study in a timely manner and none involved failure to interpret or misdiagnosis when using of POC ultrasound.

## INTRODUCTION

The use of point-of-care (POC) ultrasound in the emergency department (ED) has dramatically expanded in recent years. Performing and interpreting ultrasound examinations at the patient’s bedside without the aid of a radiologist or sonographer has become commonplace for emergency physicians (EP) and is now fully integrated into residency training.^1^,^2^ Improved patient safety and decreased time to definitive care are drivers of this dramatic expansion in use of POC ultrasound.^3^–^5^

With any change in medical practice, the opportunity arises for lawsuits related to the usage or failure to use this new practice, such as with the use of tissue plasminogen activator in thrombotic stroke.^6^ Malpractice claims are a costly reality in the healthcare system, with emergency medicine (EM) considered to be one of the higher-risk specialties. The risk of a lawsuit for an EP is approximately 7.5% each year with the projected risk of claim over a typical career being between 75% and 99%.^7^ This is an issue that affects every EP who works clinically.

With the increasing use of POC ultrasound in the ED, there is potential additional legal risk to a practicing EP. Malpractice risk to an EP may stem from failure to perform an adequate ultrasound study, failure to interpret ultrasound findings accurately, and misdiagnosis.^8^ Some EPs may choose to forgo POC ultrasound to decrease this perceived risk or to shift potential risk onto consulting services. However, POC ultrasound has become so widely integrated into the practice of EM, the failure to integrate ultrasound into practice may lead to increased legal risk for clinicians.

A previous study on this topic by Blavais et al.^9^ analyzed 659 available records for lawsuits related to POC ultrasound over a 20-year period from 1987–2007. They identified no cases related to performance or interpretation of POC ultrasound and one case related to alleged failure to perform POC ultrasound. The aim of this study was to continue this previous work to quantify and characterize lawsuits related to EPs performing POC ultrasound. We hypothesized that given the increased use and scope of practice of POC ultrasound in EM since the previous study, the current legal risk of *not* using POC ultrasound when it may be indicated may be significant for EPs and departments.

## METHODS

### Study Design/Setting

This is a retrospective review of the Westlaw database (“ALLCASES”) for reported decisions in state and federal malpractice cases involving POC ultrasound. The Westlaw database is a repository of state and federal case law, state and federal statutes, public records and other secondary information sources. It is one of the main search engines used by legal professionals for scholarly and professional work. This study was approved by the institutional review board and the requirement for informed consent was waived.

### Study Protocol

We reviewed the Westlaw database “ALLCASES” for published case law in the U.S. from January 2008 through December 2012, including federal and state decisions. Boolean search terms included “ultrasound” and “sonography” with any suffix. These terms were searched within 250 words of “emergency” with any suffix and within 10 words of “physician” or “doctor.” The search was designed and conducted by an academic professor of law (MM) with database assistance provided by a law student (NWB).

EPs with emergency ultrasound fellowship training reviewed records that were identified through the search (LS, KO). Cases were included if a physician was accused of misconduct, the patient encounter was in the ED, the interpretation or failure to perform an ultrasound was discussed to any degree and the application was within the American College of Emergency Physicians (ACEP) core ultrasound applications (trauma, intrauterine pregnancy, abdominal aortic aneurysm, cardiac, biliary, urinary tract, deep vein thrombosis, soft-tissue/musculoskeletal, thoracic, ocular, procedural guidance).^1^ Because Blaivas et al. identified one case in which an EP was named for failure to perform a study that fell within his scope of practice, methods were designed to include any ultrasound examination that could have been or was performed by a treating EP. We included cases involving ultrasound examinations performed or ordered through a radiology department that are within the scope of ACEP core emergency ultrasound applications. The inclusion criteria were broad with the intent of including cases where an EP did or could have performed a POC ultrasound.

We recorded a basic narrative of the case, the examination type involved, the department that performed the examination, and a broad category of the type of allegation (misdiagnosis, failure to interpret, failure to perform, failure to perform in a timely manner). Discrepancies were discussed between the two reviewers to reach a consensus and full consensus was reached between the two reviewers. An *a priori* plan to include a third reviewer to review discrepancies was deemed not necessary.

## RESULTS

We identified 120 records matching initial search criteria, and seven of these cases met the inclusion criteria. Two out of seven of these cases were identified by the two reviewers using the *a priori* search criteria, which upon further review were outside of the scope of the ACEP core ultrasound applications. One of these cases involved a patient with multiple bee stings, who was later found to have an ocular foreign body. Although ocular ultrasound is within the ACEP core applications, detection of intra-ocular foreign body is not. The other case involved an elderly male patient who presented with dyspnea. His final diagnosis was acute mitral valve insufficiency and the delay in obtaining an echocardiogram was discussed in the narrative as being central to his death. Identification of acute valvular insufficiency is not within the scope of POC basic cardiac ultrasound examination. The remaining cases identified are detailed in Table.

None of the cases identified were performed as POC ultrasound studies; therefore, no cases resulted from misdiagnosis with POC ultrasound or failure to interpret a POC ultrasound examination. However, all cases involved ultrasound examinations that were within the scope of EM and were ACEP emergency ultrasound core applications. All of the cases involved failure to perform a complete ultrasound study or failure to perform in a timely manner. The most common examination type was a lower extremity venous ultrasound examination (n=3). The majority of cases involved a patient’s death (n=4).

## DISCUSSION

The cost of malpractice litigation involving physicians is high. In addition to the actual indemnity payments, the cost of defending the 80% of lawsuits in which no payment is made is borne by all physicians and hospital systems through insurance premiums and defensive medicine practices that drive up national healthcare costs.^7^,^10^,^11^ As the practice landscape changes with new technologies, there is potential for legal risk to clinicians for use or failure to use newly available treatment or diagnostic modalities.

This study used available data to characterize lawsuits related to the use of POC ultrasound by EPs. We designed the study to identify any potential cases where an EP performed or could have performed a POC ultrasound examination. From 2008 through 2012, there were five lawsuits documented in the Westlaw database on this topic and in none of these cases did an EP perform a POC ultrasound examination. There have been no documented cases of misinterpretation or missed diagnoses when using POC ultrasound by an EP. Of the identified cases, all cases relate to not performing a study or not obtaining a study in a timely manner. With increasing use of bedside ultrasound and mandatory ultrasound training and assessment of competency during residency training, potential for malpractice lawsuits exists for not performing POC ultrasound examinations or not performing them in a timely manner. The results from our study are limited to support this argument.

Of the reported cases, two involved ultrasound examinations that were performed by a radiology department. EPs could have performed these at the bedside as they are within the scope of ACEP core emergency ultrasound applications. These cases are of interest because of the deferral of risk.

As the use of POC ultrasound increases in EDs nationwide, steps to ensure responsible use are warranted. It is crucial to maintain a robust, ongoing ultrasound education program and quality assurance program at every institution to ensure adequate image acquisition and interpretation. Offering timely feedback and continuing education to physicians performing ultrasound examinations will improve the quality of their studies and decrease errors. The appropriate indications for POC ultrasound, as well as sound, consistent documentation should be emphasized. Particular attention should be paid to communicating the limited and focused scope of POC ultrasound to the patients, their families, and other providers.

## LIMITATIONS

Our study has several limitations, including its retrospective nature. Given the small number of identified cases, it is difficult to approximate definitively any measure of risk to EPs using POC ultrasound. Cases settled out of court, cases with unreported decisions, or cases otherwise not publicly available (i.e. private negotiations, arbitration, sealed records, etc.) were not captured in the Westlaw database, leading to a selection bias. This private information, unfortunately, cannot be captured in any publicly available database. Our findings are representative, however, of one major legal database in the United States. The time from ED visit to court verdict or public documentation of legal proceedings may have limited our success in capturing recent cases.

The output from the Westlaw database is limited, qualitative information with each case narrative providing varying levels of detail. Information regarding the ultrasound skills of the EP, access to bedside ultrasound, the level of facility support, other barriers present to performance of ultrasound, or the medical decision making process of the physician is not available in any standardized way within the reports. Therefore, we made assumptions that these EPs could have performed the given ultrasound examination at their facility. In order to adapt the qualitative information available in the cases, we attempted to minimize subjective inferences. Therefore, we cannot comment on why ultrasound was not used in each case.

## CONCLUSION

From 2008 to 2012, the Westlaw database reported no judicial decisions against an EP performing POC ultrasound. The database reports five cases related to failure to perform an ultrasound examination that was within the scope of ACEP core emergency ultrasound applications in a timely manner. Further analyses using other legal data sources and insurance claim data are desired and further work is necessary to confirm these preliminary findings.

## Figures and Tables

**Table:** Summary of cases involving emergency physicians and point-of-care ultrasound.

Case	Case summary	Examination type	Performing department	Allegation
1	Middle-aged female presented with calf pain. Ultrasound study reported to be negative. Patient had fatal pulmonary embolism. (2012 WL 1100657 [Ohio App. 7 Dist])	DVT	Radiology	Failure to perform complete examination
2	Teen-aged female presented with calf pain, palpitations and pre-syncope. EKG and chest x-ray normal. Patient died of massive pulmonary embolism. (2012 WL 1605709 (La.App. 5 Cir.), 11–1006 [La.App. 5 Cir. 5/8/12])	DVT	Not performed	Failure to perform
3	Teen-aged boy presented after motor vehicle collision. No abdominal imaging was performed. Patient was discharged and died that night at home with liver laceration and hemoperitoneum. (721 S.E.2d 238)	FAST	Not performed	Failure to perform
4	Adult female presented with abdominal pain. Right upper quadrant ultrasound scheduled next day. Positive for cholecystitis. Alleged delay in diagnosis prolonging hospitalization and causing complications. (2009 WL 2473514)	RUQ	Radiology	Failure to perform in a timely manner
5	Adolescent male 8 days status post knee arthroscopy presented with chest pain. Diagnosed with pleurisy and discharged. Patient subsequently died of bilateral pulmonary emboli. (2012 WL 5910796)	DVT	Not performed	Failure to perform

*DVT,* deep vein thrombosis; *FAST*, Focused Assessment with Sonography in Trauma; *RUQ,* right upper quadrant

* Westlaw citations in parentheses.
